# Assaying sarcoplasmic reticulum Ca^2+^-leak in mouse atrial myocytes

**DOI:** 10.52601/bpr.2023.230044

**Published:** 2024-10-31

**Authors:** Fan Xu, Jing-Jing Li, Eric Yang, Yi Zhang, Wenjun Xie

**Affiliations:** 1 School of Life Science and Technology, Xi'an Jiaotong University, Xi'an 710061, China; 2 Department of Cardiology, First Affiliated Hospital of Xi'an Jiaotong University, Xi’an 710061, China; 3 Department of Physiology and Pathophysiology, School of Basic Medical Sciences, and Key Laboratory of Environment and Genes Related to Diseases, Ministry of Education, Xi'an Jiaotong University Health Science Center, Xi'an 710061, China

**Keywords:** Atrial myocyte, Sarcoplasmic reticulum, Ca^2+^ leak, Confocal images, Ca^2+^ measurement

## Abstract

More and more studies have suggested an essential role of sarcoplasmic reticulum (SR) Ca^2+^ leak of atrial myocytes in atrial diseases such as atrial fibrillation (AF). The increasing interest in atrial Ca^2+^ signaling makes it necessary to develop a more accurate approach for Ca^2+^ measurement in atrial myocytes due to obvious differences between atrial and ventricular Ca^2+^ handling. In the present study, we proposed a new approach for quantifying total SR Ca^2+^ leak in atrial myocytes with confocal line-scan Ca^2+^ images. With a very precious approximation of the histogram of normalized line-scan Ca^2+^ images by using a modified Gaussian distribution, we separated the signal pixel components from noisy pixels and extracted two new dimensionless parameters, *F*_signals_ and *R*_signals_, to reflect the summation of signal pixels and their release components, respectively. In the presence of tetracaine blocking SR Ca^2+^ leak, the two parameters were very close to 0, and in atrial myocytes under normal conditions, the two parameters are well positive correlative with Ca^2+^ spark frequency and total signal mass, the two classic readouts for SR Ca^2+^ leak. Consistent with Ca^2+^ Spark readouts, the two parameters quantified a significant increase of SR Ca^2+^ leak in atrial myocytes from mice harboring a leaky type 2 ryanodine receptor mutation (RyR2-R2474S^+/−^) compared to the WT group. Collectively, this study proposed a simple and effective approach to quantify SR Ca^2+^ leak in atrial myocytes, which may benefit research on calcium signaling in atrial physiology and diseases.

## INTRODUCTION

Ca^2+^ plays a pivotal role in cardiac physiological activities including contraction (Bers 2002; Szedlak *et al.* 2023). In cardiac myocytes, the sarcoplasmic reticulum (SR) serves primarily as the intracellular Ca^2+^ store providing most of the contractile Ca^2+^ for the myofilaments (Bers 2002; Lu *et al.* 2020). Ca^2+^ release from the cardiac SR is gated by type 2 ryanodine receptors (RyR2), the main SR Ca^2+^ release channels located on the membrane of junctional SR cisternae, which generates multiscale and multimodal cytosolic Ca^2+^ signals in the cardiac myocytes to mediate the cellular activities (Cheng and Lederer 2008; Xie *et al.* 2010). Dysfunctional gating of RyR2 due to stress modification or defective mutation of the channels will cause extra SR Ca^2+^ release in diastole that is termed as “SR Ca^2+^ leak”, which was first recognized in ventricular myocytes as an essential contributing factor to various ventricular pathological processes, such as heart failure and catecholaminergic polymorphic ventricular tachycardia (CPVT) (Huang *et al.* 2021; Marks 2023; Miotto *et al.* 2022; Mohamed *et al.* 2018; Steinberg *et al.* 2023). In recent years, more and more studies including ours have also revealed such SR Ca^2+^ leak in atrial myocytes promoting atrial fibrillation (AF) (Liu *et al.* 2020; Shan *et al.* 2012; Shoemaker *et al.* 2022; Tarifa *et al.* 2023; Zhang *et al.* 2021). With increasing interest in SR Ca^2+^ leak in atrial myocytes, to develop more accurate approach for atrial Ca^2+^ measurement is required due to obvious differences between atrial and ventricular Ca^2+^ handling (Bootman *et al.* 2011; Walden *et al.* 2009).

Since the discovery of the Ca^2+^ spark in 1993, the elementary intracellular Ca^2+^ event in cardiac myocytes has brought enormous information in deciphering the SR Ca^2+^ handling (Brochet *et al.* 2012; Cheng and Lederer 2008). In the past two decades, the spontaneous Ca^2+^ spark properties (frequency, total signal mass, *etc*.) were widely used as a readout to assess SR Ca^2+^ leak in ventricular as well as atrial myocytes (Huang *et al.* 2021; Lu *et al.* 2022; Shan *et al.* 2012; Zhang *et al.* 2021). However, Ca^2+^ sparks reflect only the components of SR Ca^2+^ release events with higher amplitude due to a cut-off for Ca^2+^ spark detection being introduced in order to exclude noise from signals (Brochet *et al.* 2011; Tomek *et al.* 2023; Yang *et al.* 2021). In fact, the total Ca^2+^ amount in subthreshold events is comparable to that of suprathreshold Ca^2+^ sparks (Brochet *et al.* 2011; Yang *et al.* 2021). Besides, compound Ca^2+^ sparks are also very common in atrial myocytes (Woo *et al.* 2003), which has also brought difficulty to the measurement of Ca^2+^ spark properties. An alternative measure of SR Ca^2+^ leak is to examine the effects of the RyR2 inhibitor tetracaine on diastolic Ca^2+^ levels in the absence of Na^+ ^ and Ca^2+^ in extracellular solutions (Mohamed *et al.* 2018; Zhang *et al.* 2021), which is, however, far from ideal due to the complex operation and non-physiological condition introduced.

In this work, we proposed two new parameters to quantify total SR Ca^2+^ leak in atrial myocytes using line-scan confocal Ca^2+^ images. These parameters showed well positive correlations with the classical readouts by Ca^2+^ sparks, while avoiding the complex process of detecting and analyzing Ca^2+^ sparks.

## MATERIALS AND METHODS

### Animals

Wild-type (WT) and CPVT-mutated RyR2-R2474S^+/−^ (R2474S) mice (Shan *et al.* 2012; Xie *et al.* 2013, 2015; Yang *et al.* 2021) were housed in a pathogen-free environment under a 12 h/12 h light-dark cycle and fed a rodent diet *ad libitum*. All strains of mice were on the same C57Bl/6 genetic background. All experimental protocols were approved by the Institutional Animal Care and Use Committee of Xi’an Jiaotong University and conformed to the Guide for the Care and Use of Laboratory Animals published by the National Institutes of Health.

### Mouse atrial myocyte isolation

Atrial myocytes were isolated from 3-month-old male WT and R2474S mice as previously described (Shan *et al.* 2012; Zhang *et al.* 2021). Briefly, after mouse was sacrificed, the heart is rapidly isolated, cannulated and subjected to Langendorff perfusion with perfusion buffer, comprised of (mmol/L): NaCl 113, KCl 4.7, KH_2_PO_4_ 0.6, Na_2_HPO_4_ 0.6, MgSO_4_ 1.2, NaHCO_3_ 12, KHCO_3_ 10, HEPES 10, taurine 30, glucose 1.5 and 2,3-Butanedione monoxime 10, for 5 min at a speed of 3 mL/min. Then, the perfusate was switched to digestion buffer (includes 0.65 mg/mL collagenase type 2 and 50 µmol/L CaCl_2_ in perfusion buffer) and perfused for another 10–15 min. After enzymatic digestion of the heart is complete (heart appears swollen, pale and flaccid), cut down the atria and tease them into small pieces in stop buffer (includes 0.65 mg/mL collagenase type 2, 0.065 mg/mL protease XIV, 15 mg/mL BSA and 50 µmol/L CaCl_2_ in perfusion buffer) and bath at 37 °C for 10 min, then use pipets to dissociate the heart tissue gently until all the large pieces are dispersed. After being separated from the enzyme by centrifuging for 4 min at 10 *g*, cells are resuspended in Ca^2+^-free Tyrode solution (in mmol/L): NaCl 137, KCl 5.4, MgCl_2_ 1.2, NaH_2_PO_4_ 1.2, HEPES 20, glucose 10, titrated to pH 7.4 with NaOH. The [Ca^2+^] was then gradually recovered to 1.8 mmol/L and atrial myocytes were kept in the solution until the use.

### Confocal Ca^2+^ imaging

Atrial myocytes are pre-loaded with 5 µmol/L fluo-4 AM for 15 min at room temperature, then washed out and kept in Tyrode solution containing 1.8 mmol/L CaCl_2_. A Leica TCS SP8 confocal microscopy with 63×/1.4 NA oil immersion objectives was used for confocal Ca^2+^ imaging. Cells were paced at 1 Hz lasting 1 min to normalize the store content. Line-scan images were acquired with the scanline along the long axis of cells at 400 lines/s for 20 s right after pacing. Scan zoom was fixed at 4.0 ( ~0.071 μm/pixel × 1024 pixels). The excitation for Fluo-4 is 488 nm, and emission is collected at 505–530 nm.

Ca^2+^ sparks were detected and analyzed using previously custom-developed software (Yang *et al.* 2021). The Ca^2+^ spark frequency and total signal mass, calculated by formula Σ((Δ*F/F*_*0*_) × *FWHM*^3^ × 1.206) (Brochet *et al.* 2011), are two classic readouts for SR Ca^2+^ leak, where Δ*F/F*_*0*_ represents the amplitude and FWHM (full width at half maximum) represents the spatial size of Ca^2+^ sparks.

### Total SR Ca^2+^ leak parameters

As it has been shown that Ca^2+^ sparks reflect only the SR Ca^2+^ release event with amplitude above the cut-off threshold (Brochet *et al.* 2011; Yang *et al.* 2021), we developed two new parameters free of Ca^2+^ spark to quantify the SR Ca^2+^ leak from the confocal line-scan Ca^2+^ images.

**Figure 1:**
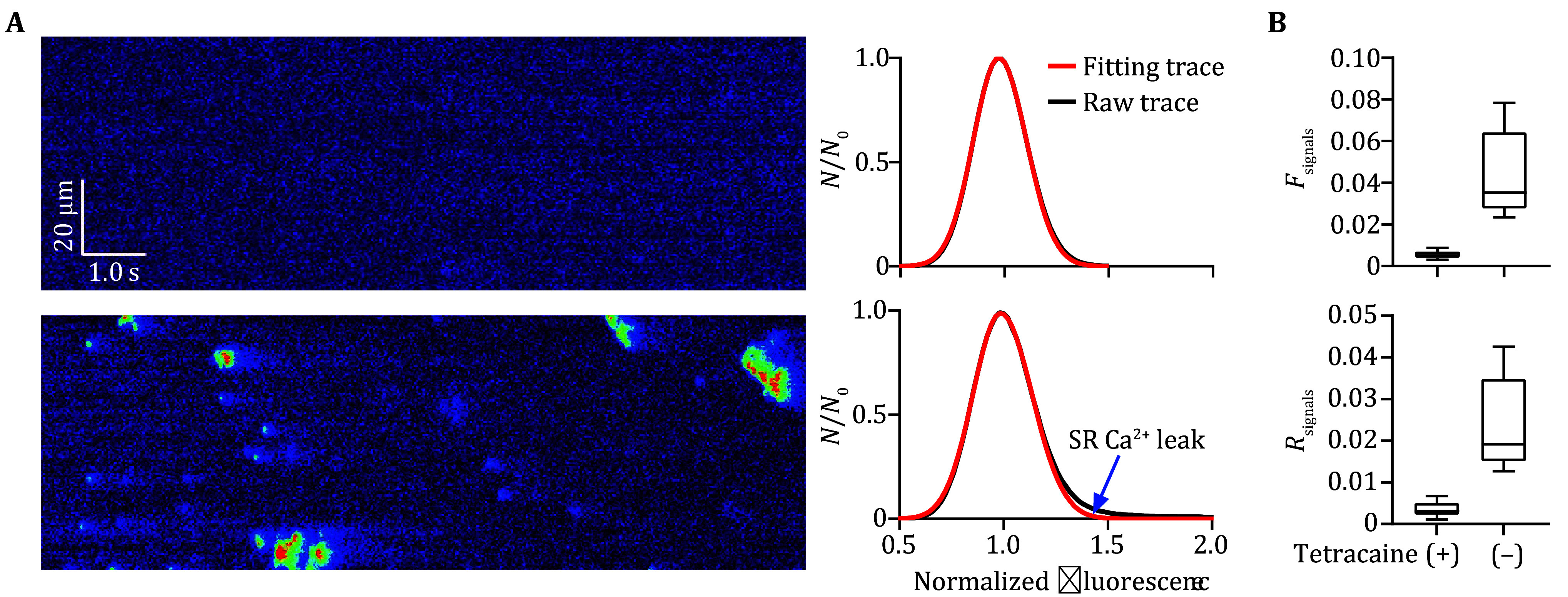
Separating Ca^2+^ signals from background noises in confocal line-scan images of atrial myocytes. **A** Typical normalized confocal line-scan Ca^2+^ images of atrial myocytes in the presence (upper) or absence (lower) of 10 μmol/L tetracaine, for which the histograms of pixel fluorescence were fitted into modified Gaussian curves in the right panel. The blue arrow indicates the difference between raw and fitting traces, which represents the signal components. **B** Statistics of *F*_signals_ and *R*_signals_ calculated from the histogram and corresponding fitting curves of line-scan Ca^2+^ images of atrial myocytes from the WT mice in the presence or absence of tetracaine, *n* = 10 atrial myocytes from three mice per group

The background noises of a confocal fluorescent image can be considered photon noise (von Wegner *et al.* 2006). After the line-scan Ca^2+^ image was normalized according to the row fluorescence without any processing for noises, a Gaussian distribution yields a good approximation to its background fluctuation (Bray *et al.* 2007; von Wegner *et al.* 2006). However, a slight rightward inclination is also notable in the background distribution (von Wegner *et al.* 2006). To better approximate the background fluctuations, we defined a modified Gaussian distribution:

1 $ N={N}_{0}{\times e}^{-\tfrac{{(f\,-\,1)}^{2}}{{{2\sigma }_{i}}^{2}}} , $

where *N*_0_ represents the peak value of the histogram of normalized fluorescent intensity *f* distribution and *σ*_*i*_ denotes the fitting standard derivation (*i* = 1 for *f* < 1 and *i* = 2 for *f* ≥ 1 with *σ*_1_ is usually a little smaller than *σ*_2_). As shown in Fig. 1A, in the presence of 10 μmol/L tetracaine blocking Ca^2+^ leak via RyR2, the histogram of normalized fluo-4 fluorescence in confocal line-scan images can be well fitted into the modified Gaussian curves. After the removal of tetracaine, there was a notable difference between the raw fluorescence histogram curve and fitting curve in the lower right part corresponding to higher fluorescent intensity, which reflects the Ca^2+^ release signals (*i*.*e*., SR Ca^2+^ leak) in the line-scan images. We defined a new dimensionless parameter to quantify the difference between both curves as follows:

2 $ {F}_{\mathrm{s}\mathrm{i}\mathrm{g}\mathrm{n}\mathrm{a}\mathrm{l}\mathrm{s}}=\int _{f=1}^{\infty }\frac{\left(H\left(f\,\right)-G\left(f\,\right)\right)}{S}fdf , $

where *H*(*f*) denotes the raw histogram curve of normalized fluo-4 fluorescence, *G*(*f*) represents the fitting Gaussian curve with Eq. 1, and *S* equals to the area of the confocal line-scan Ca^2+^ image.

Notably, *F*_signals_ reflects the summation of signal fluorescence, including both rising and declining phases of the signal pixels, which may bring errors due to overestimation of release signals. To exclude the declining phase of the signals, we calculated the temporal difference for the normalized line-scan Ca^2+^ images:

3 $ \Delta f\left(x,t\right)=f\left(x,t + \Delta t\right)-f  (\mathrm{x},t) , $

where Δ*t* equals to the scan time for each line.

Then, pixels in lines-scan images with positive Δ*f* (*x*, *t*) were counted up for histogram statistics and following Gaussian fitting. Thus, we defined another new dimensionless parameter corresponding to the summation of the rising phase of signal fluorescence as

4 $ {R}_{\mathrm{s}\mathrm{i}\mathrm{g}\mathrm{n}\mathrm{a}\mathrm{l}\mathrm{s}}=\int _{f = 1}^{\infty }\frac{\left(H'\left(f\;\right)-G'\left(f\;\right)\right)}{S}fdf , $

where *H’*(*f* ) denotes the fluorescent histogram curve of pixels with positive Δ*f* (*x*, *t*) and *G’*(*f*) represents the fitting Gaussian curve.

The new parameters *F*_signals_ and *R*_signals_ were tested for their effect in quantifying SR Ca^2+^ leak in atrial myocytes by comparing with Ca^2+^ spark frequency and total signal mass.

### Statistics

Data are presented as mean ± SD as well as individual points. Statistical analyses were carried out using Prism software version 9.0 (GraphPad Software Inc.). For continuous variables, statistical analysis was performed using a two-tailed unpaired student’s *t-*test. Differences were considered statistically significant at *P* < 0.05. All tests were 2-sided.

## RESULTS AND DISCUSSION

Intracellular Ca^2+^ plays a pivotal role in the cellular electricity and excitation-contraction coupling in atrial myocytes myocytes (Nattel *et al.* 2008). Abnormal Ca^2+^ handling in atrial myocytes has been closely associated with atrial diseases such as AF (Nattel *et al.* 2008). Similar to ventricular myocytes, the confocal fluorescent Ca^2+^ imaging has been the most commonly used approach for the measurement of intracellular Ca^2+^ signals in atrial myocytes. A typical confocal fluorescent image is formed by background noises and signals. To separate the signal pixels from the noisy ones in the line-scan Ca^2+^ images of atrial myocytes, we defined a modified Gaussian function to fit the histogram of fluorescent background fluctuations. With 10 μmol/L tetracaine blocking RyR2, the main Ca^2+^ channels in the SR of atrial myocytes, no signal but noises existed in the fluo-4 fluorescent images, in which the raw fluorescent histogram traces were well fitted into defined Gaussian curves, while Ca^2+^ signals as well as obvious differences between both traces occurred in the Ca^2+^ images in the absence of tetracaine (Fig. 1A). Correspondingly, the two new parameters calculated from the difference between raw histogram trace and fitting curve, *F*_signals_ and *R*_signals_, were almost equal to 0 in case of complete inhibition of SR Ca^2+^ release signals with tetracaine and significantly increased in the absence of tetracaine (Fig. 1B). These data suggested that the present processing is effective to distinct Ca^2+^ signal components from background noises in the line-scan fluorescent Ca^2+^ images.

**Figure 2:**
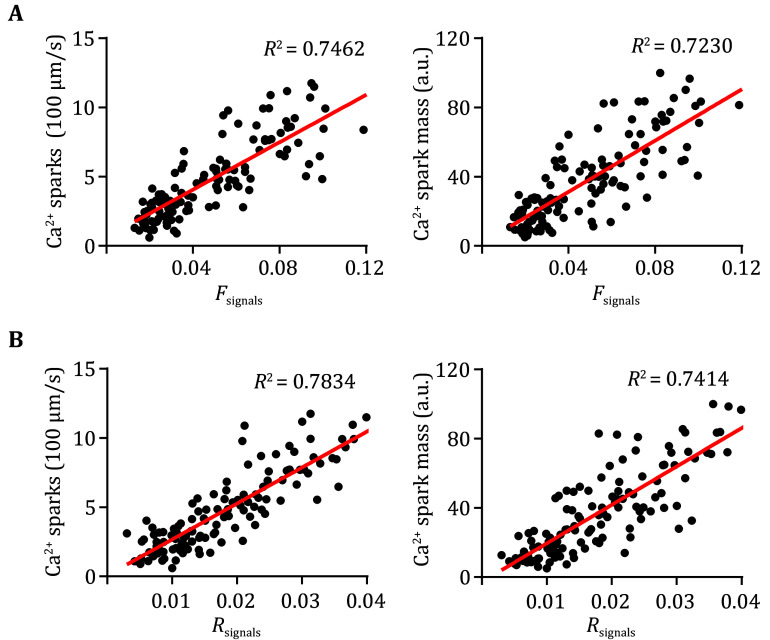
The correlations between the new parameters *F*_signals_ (**A**) and *R*_signals_ (**B**), and Ca^2+^ spark readouts. Line-scan Ca^2+^ images were processed for Ca^2+^ spark detection and new parameters calculation, and then correlation analyses were performed between the new parameters and Ca^2+^ spark readouts, *n* = 136 atrial myocytes isolated from 10 mice (5 WT and 5 R2474S)

**Figure 3:**
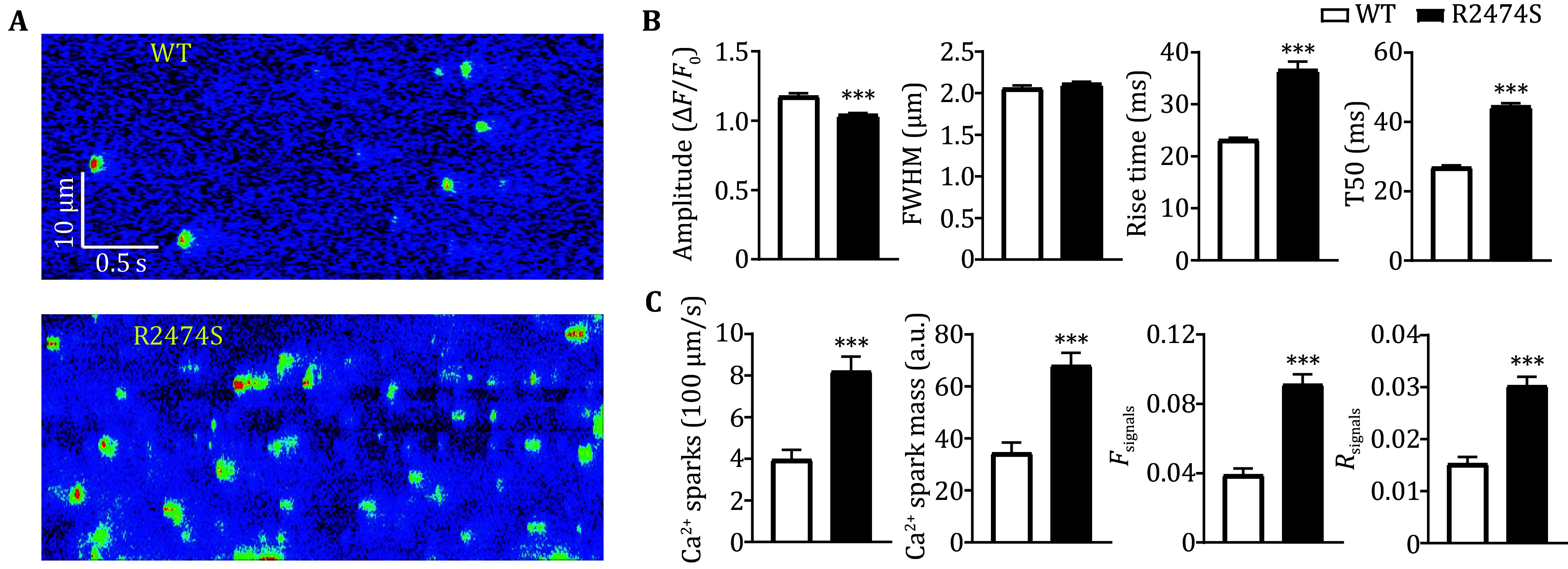
Comparison of SR Ca^2+^ leak in atrial myocytes from WT and R2474S mice with the new parameters and Ca^2+^ spark readouts. **A** Representative confocal line-scan Ca^2+^ images acquired from atrial myocytes of the WT and R2474S mice. **B** Statistics of spatiotemporal characteristics of Ca^2+^ sparks in atrial myocytes of both groups, *n* > 1000 events/group. Statistical significance was determined by unpaired *t-*test. **C** Statistics of new parameters as well as Ca^2+^ spark readouts in atrial myocytes of both groups. For the WT group, *n* = 45 cells from 5 mice; for the R2474S group, *n* = 30 cells from 5 mice. Statistical significance was determined by unpaired *t-*test

Accumulating evidence proved increased SR Ca^2+^ leak in ventricular as well as atrial myocytes essentially contributing to various cardiac diseases, such as CPVT and AF (Dridi *et al.* 2020; Houser 2023). Several approaches have been developed to quantify the SR Ca^2+^ leak in cardiac myocytes, among which Ca^2+^ spark characteristics are the most commonly used readouts. To further verify if the two new parameters can effectively reflect the SR Ca^2+^ leak in atrial myocytes, we performed the correlational analyses between them and Ca^2+^ spark frequency and total signal mass. More than 100 atrial myocytes from WT and R2474S mice were loaded with fluo-4 AM and conducted confocal line-scan Ca^2+^ imaging. As the Ca^2+^ release events are more active in atrial myocytes than ventricular myocytes (Shan *et al.* 2012; Walden *et al.* 2009), we use a recently developed threshold-free approach (Yang *et al.* 2021) to detect as many Ca^2+^ sparks, especially those with weaker amplitude. As shown in Fig. 2, both the two Ca^2+^ spark-free new parameters, *F*_signals_ and *R*_signals_ showed very good positive correlations with the Ca^2+^ spark frequency and total signal mass. R2474S mice harbored a well-known “leaky” RyR2, which displayed both increased AF susceptibility and SR Ca^2+^ leak in atrial myocytes (Shan *et al.* 2012). Consistent with previous results, the Ca^2+^ spark frequency and total signal mass in atrial myocytes from R2474S mice are 1.94 and 2.05 fold of that in WT groups (Figs. 3A–3C). A similar conclusion was achieved by using the new parameters, *F*_signals_ and *R*_signals_, which are 2.41 and 1.96 folds in the R247S group than in the WT group. These results proved that our newly developed two parameters are effective in quantifying SR Ca^2+^ leak in atrial myocytes and can be used as readouts to distinguish atrial myocytes under normal and pathological conditions.

As the elementary SR Ca^2+^ release event in cardiomyocytes, Ca^2+^ spark has been commonly used as readouts to quantify SR Ca^2+^ release (Brochet *et al.* 2011; Shan *et al.* 2012; Zhang *et al.* 2021). However, the spontaneous Ca^2+^ sparks are usually more frequent in atrial myocytes than in ventricular myocytes (Shan *et al.* 2012). For example, in atrial myocytes from R2474S mice, there exist plenty of compound Ca^2+^ sparks as well as lower-amplitude Ca^2+^ sparks (Fig. 3A), which bring out great difficulties for precise detection and characterization of these events. Besides, the temporal characteristics, including rise and decay time, of Ca^2+^ sparks are obviously greater in the R2474S group compared to the WT group (Fig. 3B), which reflects a prolonged release or multiple release even in the decay phase in R2474S atrial myocytes (Brochet *et al.* 2011). Obviously, these post-peak leaky components were ignored with Ca^2+^ spark readouts. Avoiding the trouble for Ca^2+^ spark detection and analyses in atrial myocytes, the two new parameters can be easily calculated. Besides, while the change rate (1.96) of *R*_signals_ is almost the same as Ca^2+^ spark frequency and total signal mass between the WT and R2474S group, the change rate (2.41) of *F*_signals_ is a little greater, which suggests that the parameter can include the prolonged SR leak components (Fig. 3C). An alternative approach for the measurement SR Ca^2+^ leak is the protocol developed by Shannon *et al*. (Zhang *et al.* 2021). However, since Ca^2+^ leak is a spontaneous opening event of a small number of RyR2, which occupy only a small part (about 5%) of the total SR Ca^2+^ load, such a huge difference between SR Ca^2+^ leak and load can also lead to inaccuracies for the fluorescent measurements. Moreover, the complex operation and non-physiological conditions introduced have also made it far from ideal.

Although our algorithm needs to fit the background noise, it’s not limited to processing images with special signal-to-noise ratios (SNR), in fact, the line-scan Ca^2+^ images in Figs. 2–3 have been acquired at the confocal microscope with SNR ranging from ~1.6 to ~3.4. While we have focused on atrial myocytes, our algorithm can also be used to detect the SR Ca^2+^ leak in other tissues, such as ventricular myocytes or skeletal muscle cells, since there is no special processing for atrial myocytes in this approach. Ca^2+^ wave is another type of SR Ca^2+^ release, formed by Ca^2+^ sparks propagating along cells. Whether our algorithm is suitable to quantify SR Ca^2+^ leak via Ca^2+^ waves calls for further assessment.

Taken together, the present study proposed a simple and effective approach for quantifying SR Ca^2+^ leak in atrial myocytes, to facilitate research on calcium signaling in atrial physiology and diseases.

## Conflict of interest

Fan Xu, Jing-Jing Li, Eric Yang, Yi Zhang and Wenjun Xie declare that they have no conflict of interest.
